# Randomized controlled trial of a smartphone app designed to reduce unhealthy alcohol consumption

**DOI:** 10.1016/j.invent.2024.100747

**Published:** 2024-05-17

**Authors:** John A. Cunningham, Alexandra Godinho, Christina Schell, Joseph Studer, Jeffrey D. Wardell, Claire Garnett, Nicolas Bertholet

**Affiliations:** a National Addiction Centre, Institute of Psychiatry, Psychology and Neuroscience, Kings College London, London, United Kingdom; b Centre for Addiction and Mental Health, Toronto, Canada; c Department of Psychiatry, University of Toronto, Toronto, Canada; d Humber River Health, Canada; e Addiction Medicine, Department of Psychiatry, Lausanne University Hospital and University of Lausanne, Lausanne, Vaud, Switzerland; f Service of Adult Psychiatry North-West, Department of Psychiatry, Lausanne University Hospital and University of Lausanne, Lausanne, Vaud, Switzerland; g Department of Psychology, York University, Toronto, Canada; h School of Psychological Science, University of Bristol, Bristol, United Kingdom

**Keywords:** Alcohol, Randomized controlled trial, Smartphone app

## Abstract

**Background and aims:**

Unhealthy alcohol use is common and causes tremendous harm. Most people with unhealthy alcohol use will never seek formal alcohol treatment. As an alternative, smartphone apps have been developed as one means to provide help to people concerned about their alcohol use. The aim of this study was to test the efficacy of a smartphone app targeting unhealthy alcohol consumption in a general population sample.

**Methods:**

Participants were recruited from across Canada using online advertisements. Eligible participants who consented to the trial were asked to download a research-specific version of the app and were provided with a code that unlocked it (a different code for each participant to prevent sharing). Those who entered the code were randomized to one of two different versions of the app: 1) the Full app containing all intervention modules; or 2) the Educational only app, containing only the educational content of the app. Participants were followed-up at 6 months. The primary outcome variable was number of standard drinks in a typical week. Secondary outcome variables were frequency of heavy drinking days and experience of alcohol-related problems.

**Results:**

A total of 761 participants were randomized to a condition. The follow-up rate was 81 %. A generalized linear mixed model revealed that participants receiving the full app reduced their typical weekly alcohol consumption to a greater extent than participants receiving the educational only app (incidence rate ratio 0.89; 95 % confidence interval 0.80 to 0.98). No significant differences were observed in the secondary outcome variables (*p >* .05).

**Discussion and conclusion:**

The results of this trial provide some supportive evidence that smartphone apps can reduce unhealthy alcohol consumption. As this is the second randomized controlled trial demonstrating an impact of this same app (the first one targeted unhealthy alcohol use in university students), increased confidence is placed on the potential effectiveness of the smartphone app employed in the current trial.

ClinicalTrials.org number: NCT04745325

## Highlights

• A smartphone app designed for people concerned with their alcohol use was compared to one with only educational material
• Change in drinks per week, heavy drinking days, and alcohol-related problems were compared 6-months after receiving the app
• Greater reduction in drinks per week among the full app compared to education only version; no other significant differences

## 1 Introduction

Unhealthy alcohol use is a leading contributor to the preventable burden of disease (Peacock et al., 2018; Saitz, 2005). While effective treatments exist for those with alcohol use disorders, most will never seek treatment – especially those with unhealthy alcohol use that is less severe compared to those with a more severe disorder (Cunningham and Breslin, 2004). In addition, there is substantial interest among people with unhealthy alcohol use in effective alternatives to traditional alcohol treatment to promote reductions in alcohol consumption (Koski-Jännes and Cunningham, 2001). These points, combined with the large public health impact of unhealthy alcohol consumption, emphasizes the importance of developing new means of promoting access to effective care.

Given this need, there have been substantial efforts to target unhealthy alcohol use in primary care settings (Kaner et al., 2018), as well as through the development of assisted self-change interventions. As technology develops, these interventions have increasingly utilized computer and Internet-based platforms (Bertholet and Cunningham, 2021). One more recent approach has been smartphone applications (apps). To-date, there have been a large number of such apps released for public use (Hoeppner et al., 2017; Rogers et al., 2017) though the majority have not been developed with reference to theory or evidence (Crane et al., 2015). Research on these interventions is limited, with inconsistent evidence of efficacy (Bertholet et al., 2023b; Colbert et al., 2020; Dulin et al., 2022; Leightley et al., 2022; Oldham et al., 2024; Philippe et al., 2022).

A recent randomized trial conducted by Bertholet et al., 2023a, Bertholet et al., 2023b did find evidence for the effectiveness of an app in university students. As the app may also have utility outside of the college environment, the present study tested the effectiveness in a sample of people with unhealthy alcohol use recruited from the general public. The **primary hypothesis** was that participants receiving the Full app would display greater reductions in typical weekly alcohol consumption (total number of drinks) between baseline and 6-month follow-up compared to participants who received the Educational only app.

## 2 Methods

The study design was a parallel group, double blinded, randomized controlled trial with allocation to two conditions. The trial was pre-registered (NCT04745325).

### 2.1 Recruitment

Participants were recruited using social media advertisements (e.g., Facebook) asking for those who were, “concerned about their drinking and are interested in participating in a study to find ways to help people who are worried about their alcohol use.” After reading a brief description of the study, prospective participants completed a screening questionnaire to assess eligibility for the trial: 1) 18 or older; 2) from Canada; 3) having an Alcohol Use Disorders Identification Test (AUDIT) score of 8 or more (Saunders et al., 1993); and 4) that they used a smartphone (iOS or Android). Potential participants meeting eligibility criteria were provided with a consent form that explained the purpose of the study and details of participant reimbursement. Participants were told that, “There are two versions of the smartphone app in this study that provide different types of information about drinking. The type of information you receive will be determined by chance (you have an equal chance of receiving either type of additional information).” Those who agreed to participate were asked to provide an email address, telephone number and their postal address. The postal addresses were checked to make sure that they were real and that the participant had not previously registered to take part in the trial. Only one participant per household was allowed to participate in the trial in order to reduce the chances of contamination between experimental condition.

Potential participants whose addresses were valid were sent a link by email to complete a baseline questionnaire. This questionnaire contained the AUDIT as well as other items (details provided below). Potential participants were excluded from participating in the trial if their AUDIT scores were <8 on the baseline survey, or if the score was >10 points different from the AUDIT score they provided on the screener questionnaire. This was done to ensure that participants were providing consistent answers across the screener and baseline questionnaires (please see CONSORT diagram for numbers excluded) (Schell et al., 2022). Participants who met this eligibility criteria and who completed the baseline survey were provided with the address to download the app and a code to unlock the app.

#### 2.1.1 Randomization, experimental groups

Participants who downloaded the app and entered the code were randomized to experimental condition and counted as part of the trial. In addition, they were provided with an Amazon.ca coupon (CAD$10) for completing the baseline survey and activating the app. Participants were also informed that they would receive another Amazon.ca coupon upon completion of the 6-month follow-up (CAD$20).

#### 2.1.2 Interventions groups

##### 2.1.2.1 Intervention group

Participants assigned to the intervention group were provided with the full smartphone app. Details of the content and theoretical basis for each module are provided elsewhere (Bertholet et al., 2023a). For the current study, the app was modified to apply to the Canadian context (e.g., standard drink size in Canada of 13.6 g of alcohol). Briefly, the app contained six modules: 1) a personalized normative feedback module that compared the participant's drinking to Canadian general population drinking norms generated from the Canadian Alcohol and Drugs Survey, 2019 (Statistics Canada, 2022); 2) a self-monitoring tool; 3) a goal setting tool; 4) a blood alcohol concentration estimator; 5) a tool to choose a designated driver; and 6) an educational module that contained information about alcohol and its consequences as well as links to other authoritative sources about alcohol. The instructions and code to download the app contained the text, “You have been randomized to receive educational information about risky alcohol use along with tools that will allow you to compare your own alcohol use to other people in the general population of Canada. Please have a look at these materials.”

##### 2.1.2.2 Control group

Participants assigned to the control group were provided with just the educational module (module 6) of the app. The instructions and code to download the app contained the text, “You have been randomized to receive educational information about risky alcohol use. Please download the app and have a look at these materials.”

### 2.2 Content of surveys

#### 2.2.1 Primary outcome measure

Number of standard drinks consumed on each day of a typical week in the past six months (assessed at baseline and six months). The number of drinks was summed across days to obtain an estimate of typically weekly alcohol consumption. A standard drink chart was provided (Canadian standard drink 13.6 g).

#### 2.2.2 Secondary outcome measures

1) Heavy drinking days - whether consumed five or more drinks on one occasion weekly or more often (derived from item three of the AUDIT; How often do you have five or more drinks on one occasion? With response options: Never, Less than monthly, Monthly, Weekly, Daily or almost daily; baseline and six months); 2) Number of alcohol-related consequences experienced in the past six months using the items developed by Wechsler et al. (1994) with one item added to assess drinking and driving (i.e., 11 items total; baseline and six months).

### 2.3 Sample size estimate

Power calculations were conducted using the Repeated Measures and Sample Size (RMASS) program (Roy et al., 2007). The power calculation was conducted for the primary outcome hypothesis (significantly larger reduction in total number of drinks in a typical week observed between baseline and 6-month follow-up for participants receiving the Full app compared with the Educational only app). Based on the relevant systematic reviews of digital interventions (Kaner et al., 2017; Riper et al., 2018), and on the results of our preliminary studies, we predict a small effect size (standardized mean difference, d = 0.20), which represents increased reductions of 2 drinks per week in intervention versus control group between baseline and 6-month follow-up. Based on our previous work we expected to retain at least 80 % of participants at 6-month follow-up (Cunningham et al., 2009). Using RMASS, we estimated the required sample size to detect a small standardized mean difference (d = 0.20) between the experimental conditions in the change in number of drinks from baseline to 6 months (i.e., the interaction between intervention condition and timepoint), with a specified 20 % attrition rate at 6 months factored into the sample size estimate produced by RMASS. We assumed an intra-class correlation (i.e., the variance in drinking data explained by the within-person correlation between baseline and follow-up drinking values) of ICC = 0.60 and assumed no random variance in the slopes. We also assumed no residual error correlations after accounting for the within-person correlation in pre-post data. Using this framework, we estimated that we would require *n* = 377 per condition (*N* = 754 total) to achieve adequate power.

### 2.4 Data analysis

The effects of the intervention on the outcomes were estimated using generalized linear mixed models (GLMM). Time (6-month versus baseline) was entered as a within-subject factor, and a random intercept was specified to model the variability in initial values of the outcome variables and to account for the baseline–follow-up correlation within participants. Intervention (Full app versus Educational only app) was entered as a between-subject factor, as well as a time by intervention interaction. The time by intervention interaction estimates how changes in the outcomes differed between the Full app and the Educational only app conditions.

Descriptive statistics were computed using SPSS 29 software (IBM Corp, 2022). All other analyses were conducted using R statistical software (v. 4.2.2) (R Core Team, 2022) and RStudio (v.2022.7.2.576) (RStudio Team, 2022). GLMMs estimating the intervention effect were fitted using the “glmmTMB” package's “glmmTMB” function (Brooks et al., 2017). The distributions of number of standard drinks and number of consequences were skewed. To choose the distribution that best fitted the data, a series of GLMMs with different distributions (Gaussian, Poisson, negative binomial with NB1 parameterization, negative binomial with NB2 parameterization) were estimated. To determine the best fitting models, the assumptions of each model were checked using the DHARMa (Hartig, 2022) package and Akaike Information Criterion (AIC) and Bayesian Information Criterion (BIC) values were estimated and compared. The result of the assumption checks and AIC and BIC values of each model are reported in Appendix 1. A negative binomial distribution with NB1 parameterization and a Poisson distribution were retained for number of standard drinks and number of consequences, respectively. A binomial distribution with a logit link was used for the binary heavy drinking at least weekly outcome. Participants who completed versus who did not complete the follow-up questionnaire were compared on baseline variables. Those who did not complete the follow-up questionnaire were significantly older, reported higher AUDIT scores, larger number of standard drinks, and more likely to report weekly heavy drinking (see Appendix 2). These differences were not significantly different between participants in the intervention and in the control conditions (all *p's* > 0.05). To reduce bias in parameter estimates due to participant attrition in GLMMs, missing data were handled with maximum likelihood estimation (Enders, 2006; Hallgren and Witkiewitz, 2013).

### 2.5 Ethics approval

The research was approved by the standing research ethics board of the Centre for Addiction and Mental Health.

## 3 Results

**Fig. 1:**
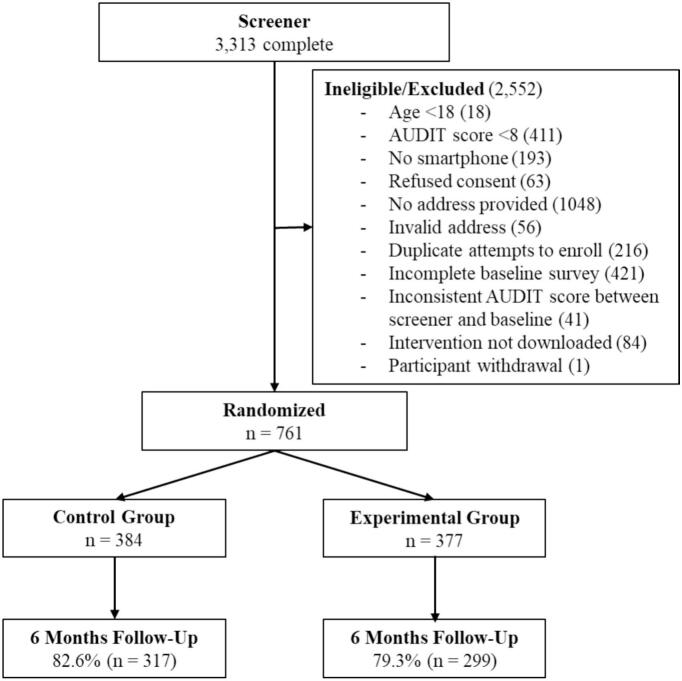
CONSORT diagram.

The study was carried out between May 27th, 2021, and April 30th, 2023. Table 1 compares baseline demographic and drinking characteristics between intervention and control conditions (no significant differences observed between condition, *p* > .05). Fig. 1 contains the CONSORT diagram for the trial. Follow-up rates were 82.6 % in the control condition and 79.3 % in the intervention condition (*p* = .269).

**Table 1:** Differences between Full app and Educational module of app only on baseline demographic and clinical characteristics.

Variable	Intervention		p
	Educational module only	Full app	
	(n = 384)	(n = 377)	
Age, mean years (SD)	42.0 (12.5)	41.7 (12.5)	0.687
Females, %	59.6	54.4	0.144
Some post-secondary or greater, %	73.2	69.2	0.231
Married/Common law, %	49.2	47.5	0.664
Full/Part-time employed, %	70.8	69.8	0.752
Household income^a^ ≤$50,000	38	36.4	0.648
AUDIT score, mean (SD)	20.3 (7.20)	20.5 (7.1)	0.739
Typical weekly drinks, mean (SD)	30.6 (20.1)	30.5 (19.5)	0.962
Drinks 5+ weekly or more often, %	70.1	67.1	0.391
# alcohol-related consequences, mean (SD)	3.8 (2.2)	3.9 (2.2)	0.882
Ever attended formal treatment for alcohol use, %	40.9	45.9	0.166
6-month follow up rate, %	82.6	79.3	0.269

Note: ^a^n = 27 reported “Don't know”

AUDIT, Alcohol Use Disorders Identification Test.

Between baseline and 6-month follow-up, participants in the intervention group used a mean of 2.75 (standard deviation 1.82) of the app modules and used the app a mean of 33.42 times (standard deviation 51.67). In the control group, 80.7 % of the participants used the educational materials module of the app and they used it a mean of 5.70 (standard deviation 7.22) times.

Results of GLMMs estimating the effect of the intervention are reported in Table 2. For the primary outcome (number of standard drinks per week), analysis showed a significant effect of time (IRR [95%CI] 0.71 [0.66, 0.76]) and a significant time by intervention interaction (IRR [95%CI] 0.89 [0.80, 0.98]), indicating that the decrease in number of standard drinks per week between baseline and follow-up was larger in the intervention (mean decrease = −10.3, standard deviation = 14.8; mean follow-up: = 19.3, standard deviation = 16.8) as compared to the control (mean decrease = −7.7, standard deviation = 17.9; mean follow-up = 21.8, standard deviation = 19.3) condition. For heavy drinking at least weekly and number of consequences, analyses showed a significant effect of time (for heavy drinking: OR [95%CI] 0.20 [0.13, 0.32]; for consequences (IRR [95%CI] 0.70 [0.64, 0.76])) but no significant time by intervention interactions. This finding indicates that heavy drinking at least weekly and number of consequences decreased significantly in the control condition between baseline and follow-up and that the decrease did not significantly differ between the intervention (heavy drinking at least weekly: percentage point decrease = −24.7; percentage follow-up = 42.4; number of consequences: mean decrease = −1.5, standard deviation = 2.5; mean follow-up = 2.4, standard deviation = 2.1) and control (heavy drinking at least weekly: percentage point decrease = −22.6; percentage follow-up = 47.5; number of consequences: mean decrease = −1.1, standard deviation = 2.1; mean follow-up = 2.7, standard deviation = 2.0) conditions.

**Table 2:** Assessment of intervention efficacy.

	# of standard drinks		Heavy drinking at least weekly		# of consequences	
	IRR	95 % CI	OR	95 % CI	IRR	95 % CI
Intervention (ref. control)	1.00	0.91, 1.10	0.79	0.48, 1.31	1.01	0.92, 1.10
Time (ref. baseline)	0.71	0.66, 0.76	0.20	0.13, 0.32	0.70	0.64, 0.76
Time by Intervention interaction	0.89	0.80, 0.98	0.91	0.50, 1.65	0.89	0.79, 1.01

Note. IRR = incidence rate ratio; OR = odds ratio; CI = confidence interval.

## 4 Discussion

Participants who received the full app reported a greater reduction in their alcohol consumption between baseline and 6-month follow-up compared to participants who only received the educational module of the app. For the secondary outcome variables, percent drinking five or more drinks on one occasion weekly or more often and number of consequences related to their drinking, there was no significant (*p* > .05) impact of receiving the full app. These results mirror the findings of the other trial examining the efficacy of the SMAART app in a sample of university students with unhealthy alcohol consumption (Bertholet et al., 2023b). Taken together, and along with the impact of the earlier version of the app employed in our pilot trial (Bertholet et al., 2019), we conclude that there is reasonable evidence for the effectiveness of this app and similar apps providing personalized feedback to have a small effect among recipients on their weekly alcohol consumption.

Limitations of this trial include a reliance on self-report data, potential for recall bias with a single follow-up assessment at six months, and a crude measure of frequency of consuming five or more drinks on one occasion. In addition, there are strengths and weaknesses to the requirement that participants provide a postal address. As a limitation, the decision may limit the external validity of the trial because some people will prefer anonymity and thus declined to participate (Romero et al., 2021). On the other hand, the requirement of a postal address (and telephone number) had a number of strengths that increased the internal validity of the study. Primarily, the requirement of a postal address allowed another way to promote the likelihood that participants could not register for the study more than once. Also, the postal address optimized the chances that only one participant per household could register in order to prevent contamination between intervention and control groups. Further, our experience is that have multiple methods of contacting participants promotes retention at follow-up. As a further limitation, the sample recruited exhibited quite high levels of alcohol concerns, having a mean AUDIT score of 20 and with >40 % reporting some form of prior treatment use, and were perhaps not the ideal target audience for a minimal intervention such as this app. Nonetheless, outside of a research setting, people with a high level of alcohol concerns or with alcohol use disorders are going to access these tools when they are made freely available (as it is generally the case for internet intervention and apps), and this may be a first step in seeking additional help. Thus, as Heather has noted, it makes conceptual sense to allow access to brief interventions among people with all levels of alcohol concerns (Heather, 1989). Finally, another item that might limit the generalisability of the findings is that the study was conducted during the COVID-19 pandemic.

## 5 Conclusion

While the impact of the SMAART app may be limited, with average reductions of 2.6 drinks per week (Canadian standard drink 13.6 g), the strengths of these type of interventions is that they can be provided at low cost and distributed widely. Thus, there is the potential for a significant public health impact (Abrams and Clayton, 2001). Further research is needed to explore ways to engage participants with the app to a greater extent, based on the assumption that greater use might be related to improved outcomes. In addition, there is a need to integrate self-help interventions such as smartphone apps into a continuum of care for those with alcohol concerns that includes access to more formal treatment options. This might encourage those who want more help than that provided by an app to seek alternative types of services. As there are multiple pathways to recovery from alcohol problems, providing a large number of options to promote reductions in alcohol consumption, including smartphone apps, may help more people address their alcohol concerns.

## CRediT authorship contribution statement

All authors have made an intellectual contribution to this research. JAC conceived the study and oversaw all aspects of the project. All authors developed the proposal and provided input on the design of the study. CS and AG conducted the trial. JS conducted the analyses. JAC wrote the original draft of the manuscript. All authors have contributed to the manuscript drafting process, have read, and approved the final manuscript.

## Declaration of competing interest

The authors have no conflicts of interests to declare.

## Figures and Tables

**Appendix 1:** Model fit and assumption check.

Distribution	AIC	BIC	Kolmogorov-Smirnov test (*p* value)	Dispersion test (p value)	Outlier test (p value)
Number of standard drinks					
Linear	11,738	11,769	<0.001	0.881	<0.001
Poisson	12,499	12,526	0.007	0.591	0.021
NB1	11,173	11,204	0.168	0.447	0.968
NB2	11,198	11,229	0.352	0.021	1.00

Heavy drinking at least weekly					
Binomial	1714	1740	0.757	0.539	1.00
Number of consequences					
Linear	5891	5922	<0.001	0.993	0.156
Poisson	5679	5705	0.598	0.094	1.00
NB1^a^	–	–	–	–	–
NB2^a^	–	–	–	–	–

Note: Models selected are in bold. ^a^Model did not converge. AIC = Akaike Information Criterion; BIC = Bayesian Information Criterion.

**Appendix 2:** Differences between participant who completed and did not complete the follow-up assessment on baseline demographic and clinical characteristics.

Variable	Follow-up participation		p
	Yes (n = 616)	No (n = 145)	
Age, mean years (SD)	40.82 (12.24)	46.28 (12.67)	<0.001
Females, %	56.8	57.9	0.852
Some post-secondary or greater, %	71.9	68.3	0.415
Married/Common law, %	48.1	49.7	0.782
Full/Part-time employed, %	70.6	69.0	0.687
Household income^a^ ≤$50,000	37.6	35.6	0.694
AUDIT score, mean (SD)	20.04 (7.03)	21.85 (7.47)	0.006
Typical weekly drinks, mean (SD)	29.53 (19.55)	35.01 (20.22)	0.003
Drinks 5+ weekly or more often, %	66.6	77.2	0.013
# alcohol-related consequences, mean (SD)	3.87 (2.20)	3.83 (2.24)	0.874
Ever attended formal treatment for alcohol use, %	43.0	44.8	0.710

Note: ^a^n = 27 reported “Don't know”

AUDIT, Alcohol Use Disorders Identification Test.
